# Permanent Antiseptic Medication

**Published:** 1885-02

**Authors:** 


*Permanent Antiseptic Medication.*—The following are the conclusions of a splendid paper of Professor Emarch, of Kiel, read at the Congress in Kopenhagen:

1. In all wounds, whether produced accidentally or by the hand of the surgeon, the most desirable result is *the healing by first intention ;*

2. This sort of healing can be always obtained by *keeping the infectious substances absolutely away from the wound, and the wounded part at rest ;*

3. As the renewal of the dressing disturbs the wounded parts, and exposes them again to the danger of infection, we can see that a *permanent medication* (that is, one which can remain *in situ* until the complete cure of the wound) is the best of all ;

4. If it is desired to avoid the renewal of the dressing before the cure has occurred, it is necessary to cleanse, close and dress the wound in such a manner that *no exciter of putrefaction nor any foreign body* remains in the wound, and that *no blood and secretion of the wound* be retained in any place.

Therefore the principal conditions of success are, (*a*) A complete *hœmostasis ;* (*b*) *To avoid that any cavity is formed* inside the wounds ; (*c*) to see that there be a *free exit* to all the secretions of the wounds ; (*d*) very accurate *asepsis* and *antisepsis ;* (*e*) the use of *compressible material for the dressings*, which *will absorb* the liquids ; (*f*) immobility of the wounded part.—*Wiener Med. Presse,—Rivista Clin. e Ter.*

